# Effect of smoking status on lung function, patient-reported outcomes, and safety among COPD patients treated with glycopyrrolate inhalation powder: pooled analysis of GEM1 and GEM2 studies

**DOI:** 10.1186/s12931-019-1112-0

**Published:** 2019-07-02

**Authors:** Donald P. Tashkin, Thomas Goodin, Alyssa Bowling, Barry Price, Ayca Ozol-Godfrey, Sanjay Sharma, Shahin Sanjar

**Affiliations:** 1 David Geffen School of Medicine, University of California at Los Angeles, Los Angeles, CA USA; 2 Sunovion Pharmaceuticals Inc., Marlborough, MA USA

**Keywords:** Bronchodilator, COPD, Glycopyrrolate, LAMA, Lung function, Patient-reported outcomes, Safety, Smoking status

## Abstract

**Background:**

Smoking is a major risk factor for COPD and may impact the efficacy of COPD treatments; however, a large proportion of COPD patients continue to smoke following diagnosis.

**Methods:**

This post-hoc analysis of pooled data from the replicate 12-week, placebo-controlled GEM1 and GEM2 studies assessed the impact of smoking status on the efficacy and safety of glycopyrrolate 15.6 μg twice daily vs placebo in patients with moderate-to-severe COPD. Data from 867 patients enrolled in GEM1 and GEM2 were pooled for analysis and grouped by smoking status (57% current smokers, 43% ex-smokers). Forced expiratory volume in 1 s (FEV_1_) area under the curve from 0 to 12 h, trough FEV_1_, forced vital capacity, St George’s Respiratory Questionnaire (SGRQ) total score, COPD assessment test (CAT) score, transition dyspnea index (TDI) focal score, daily symptom scores, and rescue medication use were assessed in current smokers and ex-smokers. Incidences of adverse events (AEs) and serious AEs (SAEs) were also assessed.

**Results:**

Treatment with glycopyrrolate resulted in significant improvements in all lung function measures, independent of smoking status. In both current and ex-smokers, changes from baseline in trough FEV_1_ were less marked in patients taking inhaled corticosteroids (ICS) than those not receiving ICS. Changes from baseline in SGRQ total score and rescue medication use were significantly greater with glycopyrrolate compared with placebo, regardless of smoking status. Changes in the CAT score, TDI focal score, and daily symptom scores significantly improved versus placebo, but only in current smokers. Improvements in patient-reported outcomes (PROs) with glycopyrrolate relative to placebo were numerically greater in current smokers than ex-smokers. The incidences of AEs and SAEs were similar regardless of smoking status.

**Conclusions:**

In this post-hoc analysis of GEM1 and GEM2, glycopyrrolate use led to significant improvements in lung function, independent of baseline smoking status; improvements were less marked among patients receiving background ICS, regardless of baseline smoking status. Improvements in PROs were greater with glycopyrrolate than placebo, and the magnitude of changes was numerically greater among current smokers. The safety profile of glycopyrrolate was comparable between current smokers and ex-smokers.

**Electronic supplementary material:**

The online version of this article (10.1186/s12931-019-1112-0) contains supplementary material, which is available to authorized users.

## Background

Tobacco smoking is well established as a major risk factor for the development of chronic obstructive pulmonary disease (COPD) [1–4]. A Swedish cohort study reported that approximately 50% of smokers eventually develop COPD [5], and according to World Health Organization data in 2012, 42% of all COPD-related deaths were attributable to tobacco smoking [6]. Unfortunately, approximately 40% of patients continue to smoke following diagnosis of COPD [7]. Smoking has been shown to reduce the efficacy of inhaled corticosteroids (ICS) in patients with asthma [8, 9] and leads to faster decline of lung function among patients with COPD treated with bronchodilator/ICS combinations [10, 11]. Smoking affects the exposure and efficacy of ICS by disrupting the histone deacetylase 2 enzyme system, increasing production of inflammatory cytokines, and activating the p38 MAPK pathway [12].

Glycopyrrolate inhalation powder (GLY; SEEBRI® NEOHALER®, Sunovion Pharmaceuticals Inc.) is a long-acting muscarinic antagonist (LAMA) approved by the US Food and Drug Administration at a dose of 15.6 μg twice daily (BID) for the long-term maintenance treatment of airflow obstruction in patients with COPD [13]. Two replicate, 12-week Phase III studies, **G**lycopyrrolate **E**ffect on sy**M**ptoms and lung function 1 and 2 (GEM1 and GEM2), demonstrated the efficacy and safety of GLY vs placebo in patients with moderate-to-severe COPD and a smoking history of ≥10 pack-years [14, 15]. The proportions of current smokers were 61% in GEM1 and 53% in GEM2. Treatment with GLY resulted in significantly improved lung function, as assessed by forced expiratory volume in 1 s (FEV_1_) area under the curve from 0 to 12 h (AUC_0–12h_) and trough FEV_1_, as well as patient-reported outcomes (PROs) [14, 15] compared with placebo. GLY was well-tolerated in both studies, with safety outcomes similar to placebo. In the primary analysis of the GEM1 and GEM2 studies, it was shown that the primary endpoint, FEV_1_ AUC_0–12h_ was improved to a similar extent in both current and ex-smokers [14, 15].

Given the significant proportion of COPD patients who smoke, and the potential for treatment efficacy to be impaired among current smokers, evaluation of the impact of smoking status on bronchodilator efficacy and safety may help to inform clinical decision-making [8]. While the primary analyses of the 2 studies briefly assessed the impact of smoking status on the primary endpoint, in this post-hoc analysis of pooled data from GEM1 and GEM2, we evaluated the impact of patients’ baseline smoking status on all efficacy and safety responses to GLY compared to placebo over 12 weeks.

## Methods

### Study design

The study designs of GEM1 (NCT01709864) and GEM2 (NCT01715298) have been published previously [14, 15]. Briefly, GEM1 and GEM2 were replicate, multicenter, double-blind, placebo-controlled studies that evaluated the efficacy and safety of GLY in patients with moderate-to-severe COPD (Additional file 1: Figure S1). Patients were randomized 1:1 to receive either GLY 15.6 μg BID or placebo delivered via the NEOHALER® device for 12 weeks; randomization was stratified according to baseline smoking status (current or ex-smoker). Ex-smokers were defined as patients who had not smoked for ≥6 months at screening. Patients’ smoking history and status, including pack-years (calculated as number of packs/day multiplied by the number of years of smoking) and date of quitting for ex-smokers, were reported using a questionnaire; smoking history was assessed at screening (pre-dose) only, while current smoking status was assessed at randomization and week 12 or at treatment discontinuation. Background ICS at stable doses and albuterol (as rescue medication) were permitted throughout the studies.

### Patients

Eligibility criteria have been published previously [14, 15]. Briefly, enrolled patients included males or females ≥40 years of age with stable, symptomatic COPD (Global Initiative for Chronic Obstructive Lung Disease [GOLD] 2011, stages 2 and 3) [16]. Patients were current or ex-smokers with ≥10 pack-year smoking history and had qualifying post-bronchodilator FEV_1_ (1 h after inhalation of ipratropium bromide 84 μg) ≥30% and < 80% of predicted normal, a FEV_1_/forced vital capacity (FVC) ratio <  0.70, and modified Medical Research Council grade ≥ 2 at the run-in visit. Patients were asked to refrain from smoking one hour before scheduled clinic visits and spirometry testing.

### Post-hoc analysis

Data from the GEM1 and GEM2 studies were pooled to compare the effect of baseline smoking status in patients receiving GLY or placebo. Evaluated endpoints included lung function (assessed by changes from baseline in FEV_1_ AUC_0-12h_, trough FEV_1_, and FVC at Week 12) and PROs (measured by changes from baseline in St George’s Respiratory Questionnaire [SGRQ] total score, COPD Assessment Test [CAT™] score, and transition dyspnea index [TDI] focal score at Week 12). Changes from baseline over 12 weeks in symptom burden and rescue medication use were assessed using data from patient diaries. Safety assessments included incidence of adverse events (AEs), serious AEs (SAEs), and serious cerebro- and cardio-vascular (CCV) AEs. Trough FEV_1_ was further analyzed based on the presence/absence of background ICS use.

### Statistical analyses

The full analysis set was used for all efficacy outcomes and included all randomized patients who received at least one dose of treatment. Changes from baseline in FEV_1_ AUC_0-12h_, trough FEV_1_, and FVC at Week 12 were analyzed using a mixed-model for repeated measures. Changes from baseline in SGRQ total score, CAT score, symptom scores, and rescue medication use over 12 weeks, and overall changes in TDI focal score at Week 12 were analyzed using a linear mixed model. The proportions of patients achieving the thresholds for minimal clinically important differences (reduction in SGRQ total score ≥ 4 units [17] and change in TDI focal score ≥ 1 [18]) were analyzed using a logistic regression model with the same terms as the linear mixed model. All models included covariates for baseline smoking status (current smoker or ex-smoker) and baseline ICS use (yes/no). Reduction in CAT score ≥ 2 [19] was also assessed by smoking status. No multiplicity adjustments were made for the post-hoc multiple comparisons.

The safety set included all patients who received at least one dose of treatment, and was used for analysis of all safety variables. Safety data were analyzed by smoking status using descriptive statistics. AEs were coded according to Medical Dictionary for Regulatory Activities version 17.0. All statistical procedures were performed using SAS® version 9.2 or higher (SAS Institute Inc., Cary, NC).

## Results

### Patient demographics and baseline characteristics

Data from 867 patients enrolled in GEM1 and GEM2 were pooled for analysis. Of these patients, 496 (57.2%) were current smokers and 371 (42.8%) were ex-smokers at the time of randomization (Table 1); less than 4% of patients had a change in smoking status during the 12-week trial. Patient demographics and disease characteristics at baseline were generally comparable between the current and ex-smoker subgroups. Ex-smokers were older (67.0 vs 60.0 years), had a longer duration of disease (6.0 vs 4.8 years), and higher rates of background ICS use (35.0% vs 24.6%) compared with current smokers. For both subgroups, there were more males (current smokers 59.1%; ex-smoker 56.9%) than females, and most patients were Caucasian (current smoker 86.7%; ex-smoker 91.1%). A greater proportion of current smokers were classified as GOLD 2 (current smoker 66.7%; ex-smoker 57.4%) vs GOLD 3 (current smoker 31.9%; ex-smoker 41.2%), and GOLD group B (current smoker 64.1%; ex-smoker 54.4%) vs group D (current smoker 34.5%; ex-smoker 43.9%).

**Table 1:** Patient demographics and disease characteristics at baseline

	Current smoker			Ex-smoker		
	GLY (*n = 251*)	Placebo (*n = 245*)	Total (*N = 496*)	GLY (*n = 186*)	Placebo (*n = 185*)	Total (*N = 371*)
Age, years, median (range)	61.0 (44, 83)	60.0 (41, 84)	60.0 (41, 84)	68.0 (43, 86)	67.0 (48, 87)	67.0 (43, 87)
Male, *n* (%)	142 (56.6)	151 (61.6)	293 (59.1)	109 (58.6)	102 (55.1)	211 (56.9)
Race, *n* (%)						
Caucasian	223 (88.8)	207 (84.5)	430 (86.7)	172 (92.5)	166 (89.7)	338 (91.1)
Black	22 (8.8)	35 (14.3)	57 (11.5)	8 (4.3)	11 (5.9)	19 (5.1)
Asian	1 (0.4)	0	1 (0.2)	2 (1.1)	3 (1.6)	5 (1.3)
Native American	2 (0.8)	2 (0.8)	4 (0.8)	0	2 (1.1)	2 (0.5)
Other	3 (1.2)	1 (0.4)	4 (0.8)	4 (2.2)	3 (1.6)	7 (1.9)
Duration of COPD, years, median (range)	4.7 (0.0, 19.0)	4.9 (0.0, 29.9)	4.8 (0.0, 29.9)	6.1 (0.0, 28.6)	6.0 (0.0, 32.6)	6.0 (0.0, 32.6)
Airflow obstruction (GOLD 2011 [16]), *n* (%)						
Mild (GOLD 1)	0	1 (0.4)	1 (0.2)	0	0	0
Moderate (GOLD 2)	166 (66.1)	165 (67.3)	331 (66.7)	111 (59.7)	102 (55.1)	213 (57.4)
Severe (GOLD 3)	83 (33.1)	75 (30.6)	158 (31.9)	72 (38.7)	81 (43.8)	153 (41.2)
Missing	2 (0.8)	4 (1.6)	6 (1.2)	3 (1.6)	2 (1.1)	5 (1.3)
Combined assessment of COPD (GOLD 2011 [16]), *n* (%)						
Group A	1 (0.4)	0	1 (0.2)	0	1 (0.5)	1 (0.3)
Group B	161 (64.1)	157 (64.1)	318 (64.1)	108 (58.1)	94 (50.8)	202 (54.4)
Group C	0	0	0	0	0	0
Group D	87 (34.7)	84 (34.3)	171 (34.5)	75 (40.3)	88 (47.6)	163 (43.9)
Missing	2 (0.8)	4 (1.6)	6 (1.2)	3 (1.6)	2 (1.1)	5 (1.3)
Estimated number of pack-years, median (range)	46.0 (10.0, 146.5)	48.0 (11.0, 184.0)	47.0 (10.0, 184.0)	46.0 (12.5, 240.0)	49.0 (10.0, 156.0)	47.0 (10.0, 240.0)
ICS use at baseline, *n* (%)	56 (22.3)	66 (26.9)	122 (24.6)	65 (34.9)	65 (35.1)	130 (35.0)
Pre-bronchodilator FEV_1_, mean (SD)						
Volume, L	1.3 (0.5)	1.4 (0.5)	1.4 (0.5)	1.3 (0.4)	1.2 (0.5)	1.2 (0.5)
% predicted	46.7 (12.8)	47.3 (12.9)	47.0 (12.8)	45.9 (12.8)	44.9 (13.5)	45.4 (13.2)
Post-bronchodilator FEV_1_, mean (SD)						
Volume, L	1.6 (0.5)	1.6 (0.5)	1.6 (0.5)	1.5 (0.5)	1.4 (0.5)	1.4 (0.5)
% predicted	55.5 (12.9)	56.3 (12.7)	55.9 (12.8)	53.2 (13.5)	52.7 (13.6)	53.0 (13.5)
FVC, L, mean (SD)						
Pre-bronchodilator	2.6 (0.9)	2.7 (0.9)	2.7 (0.9)	2.6 (0.8)	2.5 (0.8)	2.6 (0.8)
Post-bronchodilator	3.1 (0.9)	3.1 (0.9)	3.1 (0.9)	2.9 (0.9)	2.9 (0.9)	2.9 (0.9)
FEV_1_/FVC, %						
Pre-bronchodilator	50.9 (10.2)	50.8 (9.9)	50.8 (10.0)	48.9 (11.0)	47.1 (10.7)	48.0 (10.8)
Post-bronchodilator	52.2 (10.2)	52.3 (9.8)	52.2 (10.0)	50.4 (11.1)	49.1 (10.8)	49.8 (10.9)

*COPD* chronic obstructive pulmonary disease, *FEV*_*1*_ forced expiratory volume in 1 s, *FVC* forced vital capacity, *GOLD* Global Initiative for Chronic Obstructive Lung Disease, *GLY* glycopyrrolate, *ICS* inhaled corticosteroid, *SD* standard deviation

### Efficacy

#### Changes from baseline in lung function

**Fig. 1:**
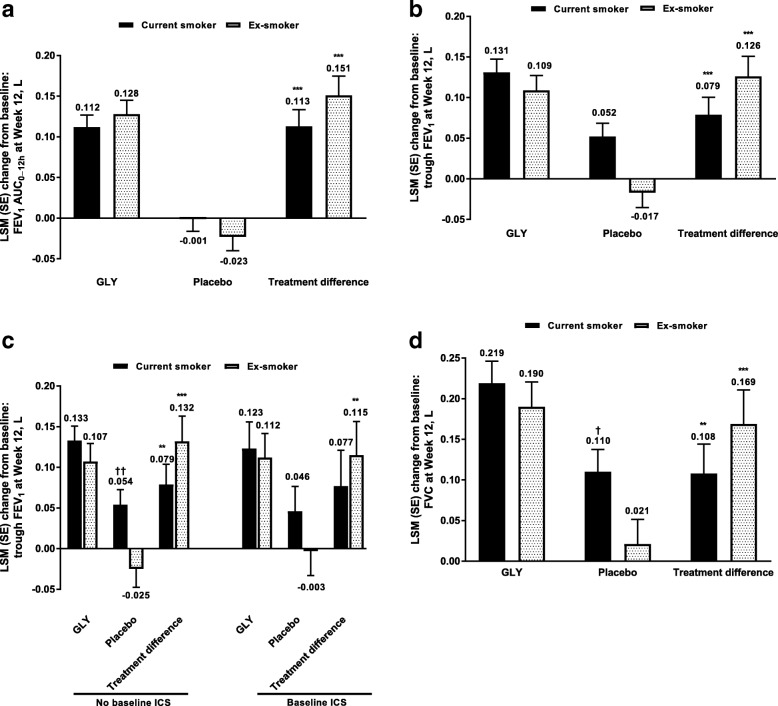
Pooled analysis of **a** FEV_1_ AUC_0–12h_, **b** trough FEV_1_, **c** trough FEV_1_ by baseline ICS use, and **d** FVC at Week 12 by smoking status (FAS). **P < 0.01, ***P **<** 0.001 vs placebo; †*P* < 0.05, ††*P* < 0.01 vs ex-smoker. *AUC*_*0-12h*_, area under the curve 0–12 h; FAS*,* full analysis set; FEV_1_*,* forced expiratory volume in 1 s; FVC*,* forced vital capacity; GLY*,* glycopyrrolate; ICS*,* inhaled corticosteroids; LSM*,* least squares mean; SE standard error

The improvement from baseline in FEV_1_ AUC_0–12h_ at 12 weeks with GLY was significant for both current smokers and ex-smokers (Fig. 1a); the placebo-adjusted improvement among patients treated with GLY was numerically greater among ex-smokers compared with current smokers. Similarly, there were significant improvements in trough FEV_1_ at Week 12 among patients treated with GLY compared with those receiving placebo, in both current and ex-smokers (Fig. 1b); the placebo-adjusted change was numerically greater in ex-smokers treated with GLY than in current smokers. In patients who did not receive background ICS, significant changes in trough FEV_1_ with GLY versus placebo occurred in both current and ex-smokers (Fig. 1c). However, in patients who received background ICS, improvements in trough FEV_1_ were significantly greater for GLY versus placebo in ex-smokers but not current smokers (Fig. 1c); this may be due to the marked differences in response observed with placebo between the smoking subgroups. Consistent with the overall improvements in trough FEV_1_, the placebo-adjusted change in trough FEV_1_ with GLY was numerically greater in ex-smokers than current smokers, whether or not patients had received background ICS.

The improvement from baseline in trough FVC with GLY was also significant in both current smokers and ex-smokers (Fig. 1d) compared with placebo; of note, FVC values were similar between current and ex-smokers and across treatment groups at baseline (Table 1). While there was no significant difference in the change from baseline in trough FVC with GLY between current and ex-smokers, the improvement among patients receiving placebo was significantly greater in current smokers than ex-smokers. Consequently, the magnitude of the placebo-adjusted improvement with GLY was larger in ex-smokers than current smokers (Fig. 1d).

#### Change from baseline in SGRQ total scores and CAT scores

**Fig. 2:**
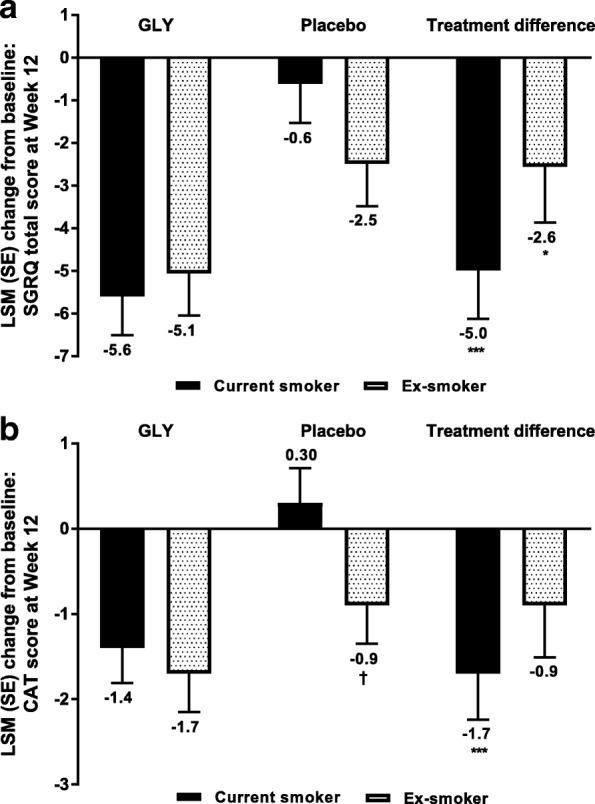
Pooled analysis of **a** SGRQ total score and **b** CAT score at Week 12 by smoking status (FAS). *P < 0.05, ***P **<** 0.001 vs placebo; †*P* < 0.05 vs smoker. CAT, COPD Assessment Test; FAS*,* full analysis set; GLY*,* glycopyrrolate; LSM, least squares mean; SE*,* standard error; SGRQ*,* St George’s Respiratory Questionnaire

The change from baseline in SGRQ total score at Week 12 was significantly greater for patients treated with GLY than those receiving placebo, regardless of their smoking status at baseline (Fig. 2a); improvements with GLY among current smokers exceeded the minimal clinically important difference (MCID; 4 units [17]) and were considered clinically significant. The placebo-adjusted improvement from baseline in SGRQ total score was numerically greater in current smokers than ex-smokers; this may be attributed, at least in part, to the improvement with placebo being greater in ex-smokers than in current smokers. Consistent with the numerically greater improvement in SGRQ total score in current smokers, odds of a current smoker being an SGRQ responder (≥4-unit reduction in total SGRQ score) were significantly greater for GLY than for placebo (odds ratio [OR]: 1.69, 95% confidence interval [CI]: 1.15, 2.48; *P* <  0.01); this was not the case for an ex-smoker (OR: 1.46, 95% CI: 0.94, 2.27; *P* = 0.089).

At Week 12, treatment with GLY was associated with a significant reduction from baseline in CAT score versus placebo in current but not ex-smokers (Fig. 2b). There was a significant difference between smokers and ex-smokers in the change from baseline in CAT score with placebo; this may have contributed to the treatment difference being smaller in ex-smokers than current smokers. Consistently, in the GLY treatment arm, the proportion of current smokers with a clinically important reduction in CAT score (≥2) at Week 12 (52.2%) was greater than the proportion of ex-smokers (44.1%); the proportions for the placebo arm were 34.2 and 42.6%, respectively.

#### Changes in TDI focal score

**Fig. 3:**
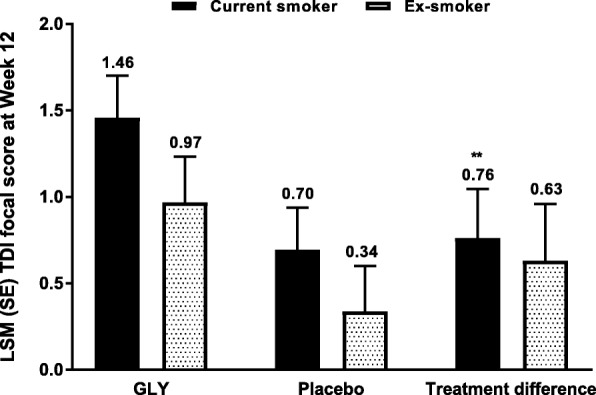
Pooled analysis of TDI focal score at Week 12 by smoking status (FAS). **P **<** 0.01 vs placebo. FAS*,* full analysis set; GLY*,* glycopyrrolate; LSM*,* least squares mean; SE, standard error; TDI*,* transition dyspnea index

For TDI focal score, the treatment difference was significant in current smokers, but not ex-smokers (Fig. 3). Placebo-adjusted improvements in TDI focal score were numerically greater among current smokers compared with ex-smokers, although the differences were not significant. The odds of achieving the MCID (≥1 [18]) were greater for GLY than placebo in both current smokers (OR: 1.82, 95% CI: 1.23, 2.70; *P* <  0.01) and ex-smokers (OR: 1.67, 95% CI: 1.06, 2.65; *P* <  0.05).

#### Changes from baseline in symptom scores

Over 12 weeks, treatment with GLY led to significant decreases in LS mean daily total symptom score compared with placebo among current smokers but not ex-smokers (Table 2). Similar results were observed for mean daytime and nighttime symptom score (Table 2). For all symptom-related PROs, the magnitude of improvement with GLY was greater in current smokers than ex-smokers.

**Table 2:** Least squares mean (standard error) change from baseline in symptom scores

	Current smoker				Ex-smoker			
Parameter	GLY	Placebo	Δ	*P* value	GLY	Placebo	Δ	*P* value
Mean total symptom score								
Daily	−1.3 (0.1)	−0.8 (0.1)	−0.5 (0.1)	< 0.001	−1.1 (0.1)	− 1.0 (0.1)	−0.1 (0.1)	0.616
Daytime	−1.1 (0.1)	− 0.5 (0.1)	− 0.6 (0.1)	< 0.001	− 0.9 (0.1)	− 0.8 (0.1)	− 0.1 (0.2)	0.746
Nighttime	− 1.2 (0.1)	− 0.8 (0.1)	− 0.5 (0.1)	< 0.01	−1.0 (0.1)	− 1.0 (0.1)	0.0 (0.2)	0.841
Mean daily cough scores	− 0.3 (0.02)	− 0.2 (0.02)	− 0.1 (0.03)	< 0.05	− 0.2 (0.03)	−0.3 (0.03)	0.1 (0.04)	0.179
Mean daily sputum production scores	−0.2 (0.02)	−0.1 (0.02)	− 0.1 (0.03)	< 0.01	− 0.2 (0.03)	−0.2 (0.03)	0.01 (0.04)	0.884
% nights with no nighttime awakening	13.7 (1.6)	7.6 (1.6)	6.1 (2.2)	< 0.01	11.8 (1.9)	13.6 (1.9)	−1.8 (2.5)	0.484
% days with no daytime symptoms	4.5 (1.2)	2.1 (1.2)	2.4 (1.5)	0.105	4.5 (1.4)	3.0 (1.3)	1.6 (1.7)	0.367
% days able to perform daily activities	6.0 (1.7)	−1.6 (1.7)	7.6 (2.3)	< 0.001	7.3 (1.9)	4.4 (1.9)	2.9 (2.6)	0.277

*GLY* glycopyrrolate, *Δ* treatment difference

#### Changes from baseline in rescue medication use

**Fig. 4:**
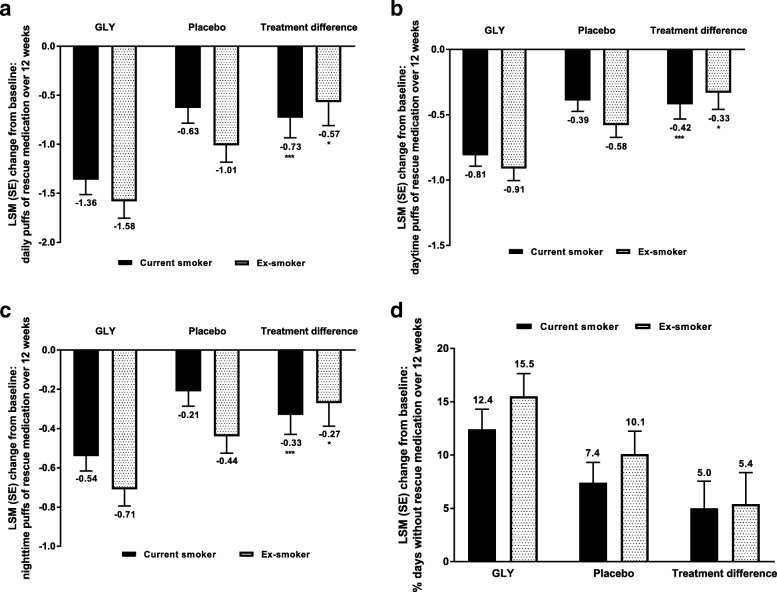
Pooled analysis of mean **a** daily, **b** daytime, and **c** nighttime number of rescue medication puffs, and **d** percentage of days with no rescue medication use over 12 weeks by smoking status (FAS). *P < 0.05, ***P **<** 0.001 vs placebo. FAS*,* full analysis set; GLY, glycopyrrolate; LSM, least squares mean; SE*,* standard error

Treatment with GLY led to significantly greater reductions in mean daily rescue medication use compared with placebo, in both current smokers and ex-smokers (Fig. 4a). GLY had a similar effect on both daytime and nighttime rescue medication use, in both current and ex-smokers (Fig. 4b-c). Overall, the magnitude of improvement in rescue medication use with GLY was greater in current than ex-smokers. The mean percentage of days with no rescue medication use was higher for GLY than placebo in both current and ex-smokers (Fig. 4d), but there were no significant differences between current smokers and ex-smokers in either treatment group.

### Safety

The overall incidence of AEs was generally similar between the treatment groups over 12 weeks, irrespective of smoking status, with the lowest incidence being among current smokers receiving placebo (39.6%) and the highest among ex-smokers receiving GLY (48.4%; Table 3).

**Table 3:** Adverse events reported by ≥2% of patients in either treatment group (safety set)

Preferred term	Current smoker		Ex-smoker	
	GLY (*n = 252*)	Placebo (*n = 245*)	GLY (*n = 186*)	Placebo (*n = 185*)
Patients experiencing ≥1 AE	116 (46.0)	97 (39.6)	90 (48.4)	83 (44.9)
COPD worsening/exacerbation	37 (14.7)	40 (16.3)	31 (16.7)	35 (18.9)
Cough	5 (2.0)	4 (1.6)	8 (4.3)	7 (3.8)
Upper respiratory tract infection	6 (2.4)	6 (2.5)	9 (4.8)	3 (1.6)
Nasopharyngitis	3 (1.2)	4 (1.6)	5 (2.7)	5 (2.7)
Headache	8 (3.2)	6 (2.5)	2 (1.1)	7 (3.8)
Oropharyngeal pain	3 (1.2)	2 (0.8)	6 (3.2)	3 (1.6)
Nasal congestion	2 (0.8)	4 (1.6)	2 (1.1)	5 (2.7)
Dyspnea	2 (0.8)	1 (0.4)	1 (0.5)	4 (2.2)
Bronchitis	2 (0.8)	1 (0.4)	1 (0.5)	5 (2.7)
Gastroenteritis viral	1 (0.4)	5 (2.0)	1 (0.5)	0

*AE* adverse event, *COPD* chronic obstructive pulmonary disease, *GLY* glycopyrrolate

The most common AE, occurring to a similar extent in both treatment arms and in current and ex-smokers, was worsening of COPD (Table 3). The incidence of cough was similar between treatments but was lower in current smokers than ex-smokers (current smokers: GLY, 2.0%, placebo: 1.6%; ex-smokers: GLY, 4.3%, placebo: 3.8%). The incidence of upper respiratory tract infections was similar among current smokers regardless of treatment (smokers: GLY, 2.4%, placebo: 2.5%), but in ex-smokers was greater with GLY (4.8%) than placebo (1.6%). In current smokers, the proportion of patients with at least one SAE was similar for GLY and placebo (4.4% vs 4.9%, respectively); in ex-smokers, the proportion was slightly higher for GLY than for placebo (4.8% vs 2.7%, respectively).

In current smokers, the incidence of serious CCV AEs was lower for GLY than for placebo (1 [0.4%] vs 5 [2.0%], respectively). There were no major adverse cardiac events (MACEs) in the GLY arm, versus 4 (1.6%) events in the placebo arm, including two cases of coronary revascularization and one each of non-fatal myocardial infarction (MI), non-fatal stroke, and heart failure requiring hospitalization. In ex-smokers, the incidences of serious CCV AEs (5 [2.7%] vs 1 [0.5%]) and MACE (3 [1.6%] vs 1 [0.5%]) were greater for GLY than for placebo. Among ex-smokers, MACE included three cases of non-fatal MI in the GLY arm, and one coronary revascularization in a patient taking placebo.

## Discussion

Smoking status is known to impact the efficacy of ICS treatment in asthma [8, 9] and leads to faster decline in patients with COPD [10, 11]. However, studies of the LAMA tiotropium [20, 21] showed non-significant long-term differences in bronchodilator response between current and ex-smokers. The results of this pooled analysis of data from the GEM1 and GEM2 studies showed that baseline smoking status did not have a significant impact on the efficacy or safety profile of GLY.

The magnitude of the effect of GLY (relative to placebo) on FEV_1_ AUC_0-12h_, trough FEV_1_, and trough FVC at 12 weeks was greater in ex-smokers than current smokers, but none of the differences between current and ex-smokers were significant. The effects of GLY were not impacted by baseline ICS use, while the impact on PROs was numerically but not significantly greater in current than ex-smokers. Conversely, in patients receiving tiotropium for 12 weeks there were numerical but not statistical improvements in FEV_1_ in current vs ex-smokers [20]. However, in the same study among current smokers, baseline airflow obstruction was less severe in the tiotropium arm than the placebo arm, whereas for ex-smokers the opposite was true. This may have accounted for the apparently greater response to tiotropium in current smokers, consistent with observations that bronchodilator responsiveness appears to be related to the degree of airflow obstruction, with patients having moderate obstruction showing greater responses to bronchodilators than those with severe obstruction by FEV_1_ criteria [22]. In contrast, in the current study, baseline lung function was similar between treatment arms and between current and ex-smokers. This may account for the slight difference in outcomes with regard to current versus ex-smokers. In the 4-year UPLIFT trial, tiotropium treatment led to greater improvement in lung function in continuing current smokers than in ex-smokers. However, these results may also have been confounded by the fact that baseline airflow obstruction was greater in ex-smokers than current smokers, and concomitant treatment with long-acting β2-agonists, ICS and methylxanthines was permitted throughout the trial [21]. Importantly, in the current analysis, improvement in lung function outcomes with placebo were greater in current smokers than ex-smokers; this may have influenced the differences in placebo-adjusted outcomes between current and ex-smokers.

The GOLD 2019 report recommended the addition of ICS to long-acting bronchodilator therapies in patients with more severe disease experiencing dyspnea or exacerbations and having high eosinophil counts [1]. Consistent with the observation that smoking can impact ICS efficacy and disease progression during treatment of patients with asthma and COPD [8–11, 23–25], in this analysis, current smokers receiving background ICS had non-significant improvement in trough FEV_1_ with GLY compared with placebo, whereas ex-smokers had a significant improvement, regardless of background ICS use; however, this was, at least in part, due to differences in lung function improvement in the placebo arm between current and ex-smokers. Provided they were not receiving background ICS, both current and ex-smokers had significant improvements in trough FEV_1_.

The most notable differences between current and ex-smokers were related to changes in PROs. While SGRQ total scores and rescue medication use were significantly improved with GLY compared with placebo in both current and ex-smokers, numerically greater improvements in placebo-adjusted SGRQ total score among current smokers compared to ex-smokers receiving GLY may be attributed, in part, to the greater improvements among ex-smokers receiving placebo. In contrast, placebo-adjusted changes from baseline in CAT and symptoms scores, as well as changes in TDI focal scores at Week 12, were significantly improved only in current smokers, but not in ex-smokers. While the differences in CAT scores between current and ex-smokers may be due to greater improvements among ex-smokers compared with smokers receiving placebo, similar differences between current and ex-smokers in placebo effects were not observed in TDI focal scores. Previous studies of tiotropium showed similar significant placebo-adjusted changes from baseline in SGRQ total score irrespective of smoking status, although numerically greater improvements were observed among current smokers compared with ex-smokers [20, 21].

The differences observed in PROs between current and ex-smokers in this analysis may have been influenced by disease severity at baseline, with patients with more severe disease having possibly ceased smoking due to worse baseline lung function. This is supported by baseline characteristics showing that more ex-smokers than current smokers had longer disease duration and were classified as GOLD 3 and GOLD group D. Another potential explanation for the greater improvements in PROs in current smokers than ex-smokers is improvements in symptoms directly driven by active smoking, such as increased mucus secretion; such improvements in symptoms directly caused by active smoking may have led to greater positive outcomes among current smokers. In addition, the central actions of nicotine in the brain, which include release of neurotransmitters such as dopamine [26], may have a positive impact on patients’ perceptions of quality of life.

The safety profile of GLY was not substantially impacted by smoking status at baseline, although there were differences in the incidences of COPD worsening, cough, and upper respiratory tract infections between current and ex-smokers and treatment arms; these may be due to differences in baseline characteristics (e.g. disease severity) between subgroups. AEs of special interest associated with anticholinergic therapies (e.g. dry mouth and dizziness) were infrequent and were similar between treatments. Overall, the AE profile of GLY was not affected by smoking status at baseline. Although CCV AEs and MACE were most frequent in ex-smokers, the incidences were low and similar to those reported for other LAMAs [27, 28].

The main limitation of this analysis is that post-hoc comparisons were not adjusted for multiplicity. Only a small number of patients stopped or started smoking during the GEM1 and GEM2 studies, which did not impact the observed outcomes. Additional prospective studies are needed to better understand the impact of smoking status on clinical outcomes with different bronchodilators available for the treatment of patients with COPD.

## Conclusions

In this post-hoc analysis of pooled data from the GEM1 and GEM2 studies, GLY treatment led to significant improvements in lung function, SGRQ total score, and rescue medication use vs placebo in both smokers and ex-smokers. Current smokers receiving background ICS therapy on top of GLY had non-significant improvements in trough FEV_1_ compared with placebo; this is consistent with previous studies that demonstrated effects of smoking on ICS efficacy. GLY treatment resulted in clinically important improvements in CAT scores, TDI focal scores, and daily symptom scores in both current and ex-smokers, with significant improvements over placebo only among current smokers. These data support the use of GLY 15.6 μg BID in patients with moderate-to-severe COPD regardless of their baseline smoking status, although the magnitude of benefit may differ between current and ex-smokers.

## Additional file

Additional file 1: **Figure S1.** Design of the GEM1 and GEM2 studies [14, 15]. ^*^Screening period was flexible, ranging between 1 to 7 days. BID, twice daily (PDF 397 kb)

## Data Availability

Sunovion Pharmaceuticals Inc. is part of a clinical trial data sharing consortium that facilitates access for qualified researchers to selected anonymized clinical trial data. For up-to-date information on data availability please visit https://www.clinicalstudydatarequest.com/Study-Sponsors.aspx and click on Sunovion.

## Abbreviations

AE: Adverse event
AUC_0–12h_: Area under the curve from 0 to 12 h
BID: Twice daily
CAT: COPD assessment test
CCV: Cerebro- and cardiovascular
CI: Confidence interval
FAS: Full analysis set
FEV_1_: Forced expiratory volume in 1 s
FVC: Forced vital capacity
GLY: Glycopyrrolate
GOLD: Global Initiative for Chronic Obstructive Lung Disease
ICS: Inhaled corticosteroids
LAMA: Long-acting muscarinic antagonist
MACE: Major adverse cardiac events
MCID: Minimal clinically important difference
MI: Myocardial infarction
OR: Odds ratio
PRO: Patient-reported outcome
SAE: Serious AE
SD: Standard deviation
SGRQ: St George’s Respiratory Questionnaire
TDI: Transition dyspnea index
