# Supplementary figures and images for: Effect of smoking status on lung function, patient-reported outcomes, and safety among COPD patients treated with glycopyrrolate inhalation powder: pooled analysis of GEM1 and GEM2 studies
Source: Respir Res. 2019 Jul 2;20:135. doi: 10.1186/s12931-019-1112-0 (PMC6604131; doi:10.1186/s12931-019-1112-0)

**12-week, multicentre, randomized, double-blind, parallel-group,  
placebo-controlled studies**

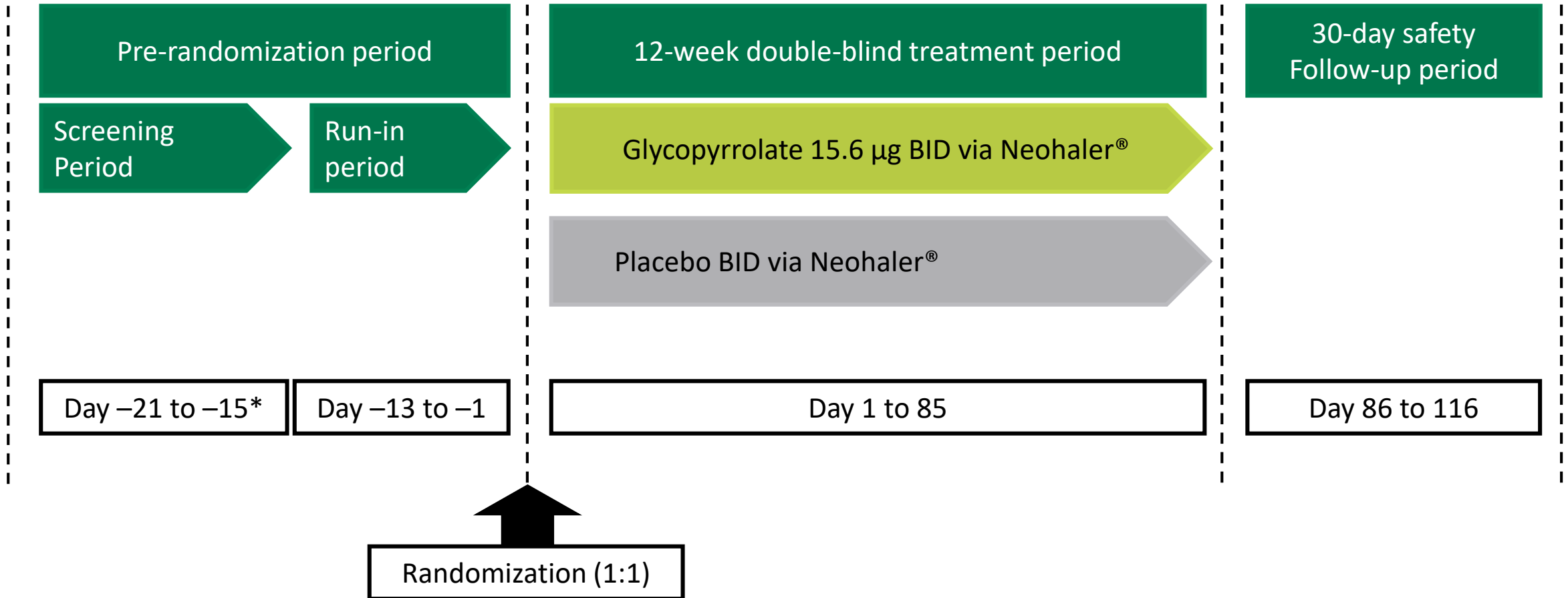

Supplement: Supplementary file 1 — Figure S1. Design of the GEM1 and GEM2 studies [14, 15]. *Screening period was flexible, ranging between 1 to 7 days. BID, twice daily (PDF 397 kb) [file 12931_2019_1112_MOESM1_ESM.pdf]
